# Spontaneous regeneration of the mandible following hemimandibulectomy for medication-related osteonecrosis of the jaw: A case report

**DOI:** 10.1097/MD.0000000000021756

**Published:** 2020-08-14

**Authors:** Shinpei Matsuda, Hisato Yoshida, Minako Shimada, Hitoshi Yoshimura

**Affiliations:** Department of Dentistry and Oral Surgery, Unit of Sensory and Locomotor Medicine, Division of Medicine, Faculty of Medical Sciences, University of Fukui, Fukui, Japan.

**Keywords:** breast cancer, elderly patient, mandible, medication-related osteonecrosis of the jaw, spontaneous regeneration

## Abstract

**Rationale:**

Medication-related osteonecrosis of the jaw decreases quality of life of patients with cancer. The debate about it continues regarding the risk factors, etiology, and treatment methods, and so on. Also, spontaneous regeneration of the mandible is clinically rare.

**Patient concerns:**

A 67-year-old woman presented to the authors’ department complaining of pain, swelling, and pus discharge from a fistula. She previously had breast cancer bone metastases and had received antiresorptive intravenous bisphosphonate.

**Diagnosis:**

The patient was diagnosed with medication-related osteonecrosis of the jaw.

**Interventions:**

She received conservative therapy with antibiotics and surgical therapy as sequestrectomy under general anesthesia; however, the lesion did not heal. Thirty months after the MRONJ diagnosis, when she was 70 years’ old, she underwent a left hemimandibulectomy without reconstruction under general anesthesia.

**Outcomes:**

Spontaneous regeneration of the mandible was observed by follow-up imaging examinations. The patient has no current subjective or objective symptoms.

**Lessons:**

This is the first case report of the spontaneous mandibular regeneration after surgery for medication-related osteonecrosis of the jaw. Additionally, this case was the oldest patient among the published mandibular regeneration cases.

## 1 Introduction

Medication-related osteonecrosis of the jaw (MRONJ) is defined as exposed bone or bone that can be probed through an intraoral or extra oral fistula in the maxillofacial region and that does not heal within 8 weeks and that occurs in patients who have received a bone-modifying agent or an angiogenic inhibitor agent and have no history of head and neck radiation therapy.^[1,2]^ MRONJ decreases quality of life of patients with cancer and/or osteoporosis. The debate about MRONJ continues regarding the risk factors, the etiology, the propriety of discontinuation of medication before dentoalveolar surgery, and the treatment methods including conservative and surgical therapy.^[1,2]^

Spontaneous bony regeneration of a segmental mandibular defect is rare phenomenon, and its mechanism is not clearly understood.^[3]^ In particular, the case of spontaneous regeneration of the mandible following hemimandibulectomy is very rare.^[4]^

In this report, the authors described the case of spontaneous mandibular regeneration following hemimandibulectomy for MRONJ, and conducted literature review on spontaneous mandibular regeneration. To the best of the authors’ knowledge, this is the first case report of the spontaneous regeneration of the mandible associated with advanced MRONJ. Additionally, this case was the oldest patient among the published cases of spontaneous mandibular regeneration.

## 2 Consent

Written informed consent was obtained from the patient for publication of the case and any accompanying images.

## 3 Case report

**Figure 1:**
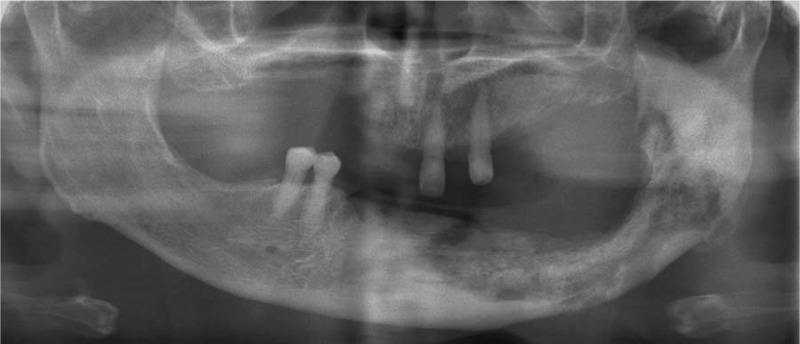
Orthopantomography 3 weeks before hemimandibulectomy.

**Figure 2:**
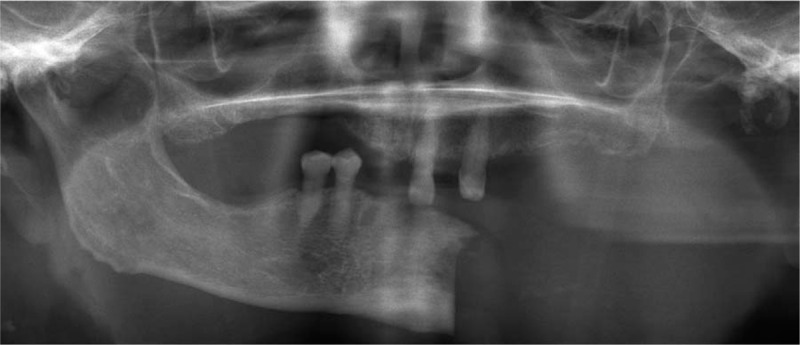
Follow-up orthopantomography 2 weeks after hemimandibulectomy.

**Figure 3:**
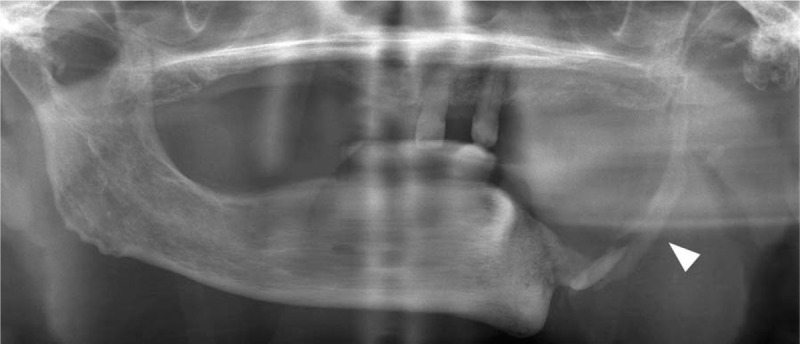
Follow-up orthopantomography 2 years after hemimandibulectomy revealed partial spontaneous regeneration of the left mandible (white arrow).

A 67-year-old woman presented to the authors’ department complaining of pain, swelling, and pus discharge from a fistula. She previously had breast cancer bone metastases and had received antiresorptive intravenous bisphosphonate (zoledronate). She was clinically diagnosed with MRONJ in the left mandibular molar region based on an American Association of Oral and Maxillofacial Surgeons’ position paper.^[1]^ She received conservative therapy with antibiotics and surgical therapy as sequestrectomy under general anesthesia; however, the lesion did not heal. A histopathological specimen examination revealed osteonecrosis of the mandible consistent with a clinical diagnosis of MRONJ. Thirty months after the MRONJ diagnosis, when she was 70 years’ old, she underwent a left hemimandibulectomy without reconstruction under general anesthesia (Figs. 1 and 2). The periosteum was preserved during resection. Thereafter, no symptoms associated with MRONJ were observed. The authors considered her lesion healed and continued with follow-up observations. Follow-up imaging examinations, such as orthopantomography and computed tomography scans, conduced 2 years after the left hemimandibulectomy revealed partial spontaneous regeneration of the left mandible (Fig. 3). She has no current subjective or objective symptoms. She can eat without using dentures and maintains good nutrition.

## 4 Review of the literature

### 4.1 Literature search strategy

Electronic searches of the PubMed were performed on 10 January 2020. A literature review was conducted based on texts published between 2003 and 2019 in this study because the first case of MRONJ was reported in 2003. The literature search strategy was as follows: ((((spontaneous[Title]) AND regeneration[Title]) AND mandible[MeSH Terms]) AND mandibular[MeSH Terms]) AND (“2003”[Date - Publication]: “2019”[Date - Publication]).

## 5 Results

Nine texts were extracted by the literature search. Twenty four patients (15 males and 9 females) were included in this review (Table 1).^[3,5–12]^ The youngest was 6 years’ old, and the oldest was 51 years’ old, and their mean age and standard deviation was 21.6 ± 12.9 years.^[3,5–12]^ The most common disease associated with surgical procedures was ameloblastoma (12 patients).^[3,5–12]^ There is no case of MRONJ in those reports. There were some cases performed immediate reconstruction, and some cases preserved the periosteum.

**Table 1:**
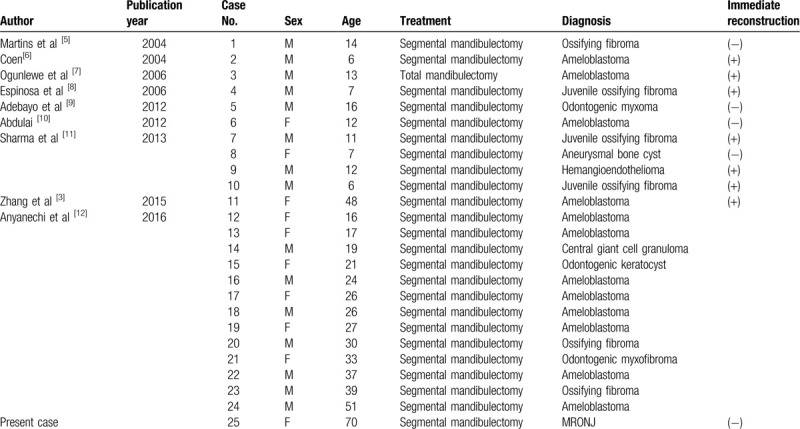
Literatures associated with spantaneous mandibular regeneration published between 2003 and 2019.

## 6 Discussion

At first, MRONJ was reported by Marx in 2003.^[13]^ However, there is room for further consideration of prevention and treatment methods of it. Conservative therapy has been accepted as the initial approach to MRONJ treatment in the latest clinical guideline by Multinational Association of Supportive Care in Cancer, International Society of Oral Oncology, and American Society of Clinical Oncology.^[2]^ On the other hand, surgical therapy may be more effective than conservative therapy for advanced cases of MRONJ; however, to date, there is no gold standard in those cases.^[14,15]^ The advantages and disadvantages of reconstruction after surgical procedures for MRONJ are also unclear, and reconstruction with a plate and screws remains challenging.^[16,17]^ Advances in the treatment and management method of MRONJ will contribute to maintain and improve the quality of life of patients with cancer and/or osteoporosis.

Spontaneous mandibular regeneration following hemimandibulectomy without reconstruction in elderly patient is very rare phenomenon. Although the mechanism and predictive factors of it is not fully understood, several predictive factors such as age, a relatively young periosteum, local infection, osteogenic progenitor cells, postoperative immobilization, and mechanical stress on the mandibular stumps, have been suggested.^[3,4,18]^ The literature review in this study clarified that spontaneous regeneration of the mandible was usually observed in young and middle aged patients.^[3,5–12]^ However, Okoturo et al suggested that increasing age might not imply a decrease in periosteal bone-regenerating potential, and the present case of this report supported that suggestion.^[18]^ However, it is necessary to consider that the patient in this case had used bisphosphonates as treatment for bone metastasis from breast cancer. Although some of cases in literature review performed reconstruction with a plate and screws accompanied by intermaxillary fixation, those were not mandatory surgical procedures for spontaneous regeneration of the mandible.^[3,5–12]^ In some reports, spontaneous mandibular regeneration was noticed by the surgeons at relatively soon after surgery, between 2 and 17 weeks.^[12,19]^ In the present case, follow-up imaging examinations 2 years after hemimandibulectomy revealed that bone regeneration occurred even in an elderly patient, and even after MRONJ treatment without reconstruction accompanied by postoperative intermaxillary fixation. Additionally, mandibular regeneration occurred from the upper edge of the remaining bone toward the coronoid process region. That indicates that bone regeneration occurred without decreasing the oral cavity volume. Therefore, the authors hypothesize that oral function including mobility of the tongue and buccal mucosa may influence spontaneous regeneration of the mandible. This case may convince clinicians to reconsider the predictors and influenced factors of spontaneous regeneration of the mandible reported by some literatures.

This case may provide important information that contributes to advances in the treatment and management method of MRONJ. In the future, long-term follow-up after surgery in MRONJ patients may clarify a new course of healing, and clarify the mechanism of spontaneous regeneration of the mandible. Furthermore, it may clarify the mechanisms and predictive factors of spontaneous bone regeneration after the segmental mandibular resection.

In conclusion, although the mechanism and predictive factors of spontaneous regeneration of the mandible is not fully understood, this case presented the new healing process for MRONJ. In the future, long-term follow-up after surgery in MRONJ patients may clarify the new information about treatment method for MRONJ.

## Author contributions

SM contributed in conception this study, and wrote original draft. HY, MS, HY revised and edited this article. All authors read and approved this study.
